# Correction: Click functionalized biocompatible gadolinium oxide core-shell nanocarriers for imaging of breast cancer cells

**DOI:** 10.1039/d2ra90119f

**Published:** 2022-12-02

**Authors:** Shifaa M. Siribbal, Shaista Ilyas, Alexander M. Renner, Sumiya Iqbal, Sergio Muñoz Vázquez, Abubakar Moawia, Martin Valldor, Muhammad S. Hussain, Klaus Schomäcker, Sanjay Mathur

**Affiliations:** Institute of Inorganic Chemistry, University of Cologne Greinstrasse 6 50939 Cologne Germany sanjay.mathur@uni-koeln.de +49 221 470 5627; Clinic and Polyclinic for Nuclear Medicine, University of Cologne Kerpenerstrasse 62 50937 Cologne Germany; Max-Planck-Institut für Chemische Physik Fester Stoffe Nöthnitzer Strasse 40 01187 Dresden Germany; Centre for Materials Science and Nanotechnology, Department of Chemistry, University of Oslo Blindern 0315 Oslo Norway; Cologne Center for Genomics (CCG), University of Cologne, Faculty of Medicine and University Hospital Cologne 50931 Cologne Germany; Center for Biochemistry, Medical Faculty, University of Cologne 50931 Cologne Germany

## Abstract

Correction for ‘Click functionalized biocompatible gadolinium oxide core-shell nanocarriers for imaging of breast cancer cells’ by Shifaa M. Siribbal *et al.*, *RSC Adv.*, 2022, **12**, 31830–31845, https://doi.org/10.1039/D2RA00347C.

The authors regret the omission of a funding acknowledgement in the original article. This acknowledgement is given below.

The authors gratefully acknowledge the financial support and infrastructure provided by the University of Cologne within the framework of UoC-Forum, iRNA-Carriers “Transformative Nanocarriers for RNA Transport and Tracking” – Advanced Concepts for Therapy and Diagnostic. We also thank the German Academic Exchange Service (DAAD) for its financial support.

The Royal Society of Chemistry apologises for these errors and any consequent inconvenience to authors and readers.

## Supplementary Material

